# Special Issue: “Advanced Research of Skin Inflammation and Related Diseases”

**DOI:** 10.3390/ijms27041830

**Published:** 2026-02-14

**Authors:** Valentina Giudice, Filomena Napolitano

**Affiliations:** 1 Hematology and Transplant Center, University Hospital “San Giovanni di Dio e Ruggi d’Aragona”, 84131 Salerno, Italy; vgiudice@unisa.it; 2 Department of Medicine and Surgery, University of Salerno, 84081 Baronissi, Italy; 3 Department of Translational Medical Sciences, University of Naples Federico II, 80131 Naples, Italy

The skin is a complex organ essential to host physiology, serving as a barrier against fluid loss, a regulator of body temperature, and a mediator of sensory perception [1]. Beyond these functions, it harbors a highly specialized immune microenvironment that is critical for maintaining tissue homeostasis, host defense, and tissue repair [2]. The disruption of this barrier can provoke inflammation and initiate a cascade of immune responses. Dysregulation of these immune mechanisms underlies a broad spectrum of inflammatory skin disorders, including eczema, dermatitis, psoriasis, and acne [3,4]. Skin inflammation arises from a complex interplay of genetic susceptibility, infectious triggers, immune dysregulation, lifestyle factors, psychological stress, and environmental exposure [5]. Despite advances in understanding disease pathogenesis, clinical heterogeneity, the absence of specific biomarkers, and variability in treatment guidelines continue to pose significant challenges for diagnosis, stratification, and management.

This Special Issue, “Advanced Research of Skin Inflammation and Related Diseases”, highlights recent advances in the pathophysiology of skin inflammation, encompassing cellular and molecular mechanisms, genetic susceptibility, cytokine signaling, the identification of potential biomarkers, and emerging therapeutic strategies.

Wang et al. developed three distinct methodologies to induce inflammatory responses in human skin biopsies using defined chemical triggers—DNCB, PMA, and ionomycin—that perturb key biological pathways implicated in the pathogenesis of inflammatory skin diseases. These ex vivo models constitute a promising translational platform for the preclinical evaluation of anti-inflammatory and immunomodulatory therapies, effectively bridging the gap between conventional in vitro systems and in vivo human studies [Contribution 1].

Sonzogni et al. demonstrated that skin fibroblasts from individuals self-diagnosed as electrosensitive comprise two distinct cellular subsets characterized by the delayed nucleoshuttling of the protein kinase ataxia telangiectasia mutated (ATM), indicating an altered cellular stress response and a heterogeneous biological basis of the condition [Contribution 2]. These findings may be relevant to inflammatory skin diseases, as impaired ATM nucleoshuttling in skin fibroblasts suggests defective stress response and DNA damage signaling pathways that can promote chronic inflammation and altered tissue homeostasis. Rella et al. demonstrated that fructose exposure increases the accumulation of advanced glycation end products (AGEs), induces the release of pro-inflammatory mediators, and activates the senescence-associated pathways p16, p21, and p53, ultimately promoting cellular senescence in skin fibroblasts [Contribution 3].

In a model of fibrotic skin disease such as Systemic Sclerosis (SSc), Napolitano et al. elucidated a key molecular pathway involving the urokinase plasminogen activator receptor (uPAR), providing new insights into disease pathogenesis and identifying potential therapeutic targets [Contribution 4]. The authors demonstrated that the increased proliferative rate of SSc dermal fibroblasts compared with healthy controls is driven by the activation of formyl peptide receptors (FPRs) and their interaction with uPAR. This interaction triggers Rac1 and ERK signaling, leading to c-Myc phosphorylation and Cyclin D1 upregulation, ultimately promoting cell cycle progression. Targeting the FPR–uPAR axis may therefore pave the way for the development of novel targeted therapies for fibrotic diseases.

Pilutin et al. showed that letrozole treatment in adult male rats induces skin structural alterations associated with oxidative stress, marked by decreased catalase expression and increased caspase-3 activation, while vitamin C co-treatment mitigates these effects, protecting against oxidative damage and apoptosis in the skin [Contribution 5].

In the search for novel biomarkers for inflammatory skin diseases, Dobrican-Băruța et al. analyzed serum levels of the alarmin triad—IL-25, IL-33, and Thymic Stromal Lymphopoietin (TSLP)—and identified IL-33 as a promising biomarker for chronic spontaneous urticaria [Contribution 6]. Choksi et al. identified potential metabolites belonging to compound classes such as bile acids, lipoxins, and phospholipids that are associated with skin disease activity in Psoriatic Arthritis [Contribution 7].

Emerging insights into inflammatory and fibrotic skin disorders have been highlighted in literature reviews. In particular, Zhou summarized the role of NLRP10 in maintaining skin homeostasis and barrier integrity, identifying this molecule as a potential therapeutic target for modulating epidermal cell death and restoring barrier function in atopic dermatitis [Contribution 8]. Grafanaki et al. highlighted overlapping pathogenic pathways shared by fibrotic disorders of the skin and lungs. Environmental stressors, such as pollution and climate change-related factors, further exacerbate these processes and should be systematically evaluated into precision therapeutic strategies [Contribution 9]. Matwiejuk et al. presented current knowledge on the association between atopic dermatitis and metabolic syndrome, highlighting a significant positive association with central obesity (measured by waist circumference) [Contribution 10]. Gu et al. highlighted macrophage plasticity as a central feature in the progression and resolution of inflammatory skin diseases. Insights into the mechanisms governing macrophage polarization and recruitment provide a conceptual framework for the development of therapeutic strategies aimed at restoring balance within the inflammatory microenvironment without broadly suppressing immune function [Contribution 11].

Finally, complementary approaches aimed at supporting skin health and resilience have gained increasing attention. Notably, Napolitano et al. investigated the effects of Nitrodi’s spring water and reported potential benefits in modulating skin cell physiology [Contribution 12].

Collectively, these studies emphasize a multi-layered view of skin disease pathogenesis, in which keratinocyte function, immune cell dynamics, fibroblast activation, systemic metabolic status, and environmental exposures intersect. Future research that bridges molecular insights with translational applications will be crucial for advancing the therapeutic strategies in both inflammatory and fibrotic skin disorders. Multidisciplinary efforts integrating dermatology, immunology, and environmental health will be essential to effectively address the complexity of these conditions.
